# When Numbers Whisper Their Names – Number Word Processing in Multi-Digit Number Magnitude Comparison in French Speakers

**DOI:** 10.1162/OPMI.a.350

**Published:** 2026-04-17

**Authors:** Roman Janssen, André Knops, Arnaud Viarouge, Elise Klein

**Affiliations:** Université Paris Cité, CNRS, LaPsyDÉ, Paris, France

**Keywords:** unit-decade compatibility effect, Sapir-Whorf hypothesis, number word syntax, number words, base-20 system, base-10 system, decimal system

## Abstract

French number words provide a unique window into the relationship between numerical cognition and language, because numbers above 60 follow a vigesimal (base-20) word structure (e.g., 72 = “60–12”). In a two-digit magnitude comparison task with sixty French native speakers, we replicated the classic unit-decade compatibility effect (UDCE; slower responses when unit and decade comparisons conflict) and within-decade effect (faster responses when decades are identical), reflecting the place-value structure of Arabic numerals. Given the French vigesimal system, we expected not only the classic UDCE and within-decade effect but also their vigesimal counterparts driven by magnitudes of number words: a unit-vigintade compatibility effect (UVCE) and a within-vigintade effect, in which pairs sharing the same decade word (e.g., “soixante” for the 60s and 70s) are processed faster than other between-decade pairs. Linear mixed models revealed both a UDCE for numbers larger than 60 and a UVCE, indicating that number words were accessed during processing. Participants also responded faster to within-vigintade items (86 vs. 95) than to between-vigintade items (76 vs. 85) and as fast as to within-decade items (82 vs. 85), indicating a verbal equivalent of the within-decade effect. This effect is unaffected by decade distance and can only be explained by access to number words so that the decades were identical (“80–6” vs. “80–15”). Overall, our data indicate that verbal representations can shape basic numerical judgments and that number processing may be more closely tied to language than previously assumed.

## INTRODUCTION

One of the central debates in cognitive psychology concerns the extent to which language shapes thought. According to the linguistic relativity hypothesis, a weaker version of the Sapir-Whorf hypothesis (Hoijer, 1954; Sapir, 1929; Whorf, 1964), a language’s grammatical and lexical properties influence how speakers perceive and conceptualize the world (Hoijer, 1954). This view contrasts with theories of cultural universality that argue that core human concepts and basic cognitive principles are not shaped by a language, but transcend linguistic boundaries (Au, 1983). A related recent proposal, rooted in the Language of Thought framework (Fodor, 1975), suggests that humans possess an internal mental language which is independent of spoken natural language (Dehaene et al., 2022). That is, according to Dehaene et al. (2022), language is a tool to express an internal Language of Thought.

In the domain of numerical cognition, a few phenomena support the idea of linguistic relativity. One line of evidence comes from indigenous tribes that have a limited vocabulary for exact numbers. The Mundurukú, an indigenous tribe in the Amazon, did not develop a full-fledged number system (Pica et al., 2004). Their language offers distinct number words for quantities up to five only. For quantities that exceed five, they use more vague quantifiers such as ‘many’, ‘really many’, or ‘two hands’. Because their language does not allow expressing exact larger quantities, their responses vary when asked to provide a verbal label for the number of dots they are presented (Pica et al., 2004). Similarly, the Pirahã, another Amazonian tribe whose language reportedly lacks exact number words beyond ‘one’, ‘two’, and ‘many’, perform poorly on tasks requiring precise quantity matching once the quantities exceed three (Frank et al., 2008; Gordon, 2004). These findings indicate that the lexical availability of exact number words may be necessary for forming consistent concepts of exact numbers beyond the subitizing range. Another line of evidence that supports linguistic relativity shows that children who speak languages with a transparent number word syntax—where the number words directly reflect the underlying numerical structure (47 = “40–7” = “forty-seven”)—have an advantage in learning how to read and write numbers (Moeller, Zuber, et al., 2015).

On the other hand, two reasons speak against linguistic relativity of numbers, supporting their language-independence: First, Arabic digits universally appear across cultures. Many numerical tasks (e.g., addition, magnitude comparison) can technically be carried out using only digit-related rules, without any need for words, just as a computer or a mental abacus works purely by processing elementary representations. Second, Pajot et al. (2025) recently argued that language merely reflects (rather than shapes) how a ‘Language of Thought’ serves in constructing numbers. Their theory posits that number concepts emerge from a language-independent internal mental syntax involving primitives like addition and multiplication.

The French language provides an interesting test bed for exploring the influence of language on the understanding of concepts in the numerical domain. While the written notation follows the canonical base-10 place-value system of Arabic digits, French number words show markedly different organization principles for numbers larger than 60. Number words above 60 follow a base-20 (*vigesimal*) system. That is, numbers from 60 to 79 share the same *vigintade* (“soixante”) and so do numbers from 80 to 99 (“quatre-vingt”). For example, the number 72 is pronounced “soixante-douze”, which translates to “sixty-twelve”. This peculiarity allows to test to which extent the linguistic properties of the French language influence the semantic processing of numbers. If language merely reflects the principles according to which a number-related ‘language of thought’ is adopted to construct numerical meaning, no traces of a vigesimal system should be observed in the processing of Arabic numbers. In contrast, if language influences the processing of the supposedly language-independent concept of Arabic numbers, we expect performance patterns that parallel the vigesimal system. The current study aimed at investigating if such vigesimal influences on two-digit number processing can be observed in French speakers by employing a classical two-digit number comparison task.

While earlier models assumed that two-digit numbers only activate a single, holistic semantic entry, a compelling line of evidence suggests the integration of digits into a multi-digit place-value system from which the numbers can be further processed. This place-value integration is prominently explored via the so-called *unit-decade compatibility effect* (UDCE; Nuerk et al., 2001; for a review see Nuerk et al., 2015). The UDCE demonstrates that not only is the overall magnitude of a two-digit number processed but also decomposed representations of the tens’ and units’ magnitudes. Specifically, participants are slower when the comparison of units and the comparison of decades lead to opposing results (e.g., 47_83 where 4 < 8 but 7 > 3) than when they lead to the same results (43_87 where 4 < 8 and 3 < 7). The UDCE is observed across presentation modalities (auditory or visual) and increases in strength as a function of the proportion of within-decade items (e.g., 45_48) that increase the importance of the unit digit in the comparison (Moeller et al., 2013) and facilitate number comparisons due to the identical decade digit which lead to faster responses (Huber et al., 2014). The UDCE is an interference effect caused by the irrelevant unit digit, whose influence must be inhibited (Nuerk et al., 2001, 2005). Thus, the UDCE is driven by the place-value structure of the two to-be-compared numbers (Nuerk & Willmes, 2005).

Crucially, the UDCE also interacts with language, particularly the syntax of number words. In languages with inverted number word structures, such as German and Dutch, where 21 is pronounced as “one-and-twenty” (unit before decade), the UDCE is larger compared to languages like English, where number word order aligns with the numerical structure (e.g., Imbo et al., 2014; Moeller, Zuber, et al., 2015; Pixner, Moeller, et al., 2011). This linguistic influence extends beyond number comparison tasks, affecting arithmetic fact retrieval (Bahnmueller et al., 2020) and carry operations in addition (Göbel et al., 2014). Moeller, Shaki, et al. (2015) further demonstrated that the UDCE varies across languages (English, German, Hebrew, and Arabic), with larger effects in German and Hebrew, where reading direction and number word order conflict, compared to English and Arabic, where they align. This suggests that number word syntax shapes the syntactic processing of Arabic numerals before their semantic magnitudes are evaluated.

One might argue that the increased UDCE in German and Dutch speakers could be due to the stronger focus on the unit digit when reading multi-digit numbers. This would mean that numbers are not directly processed linguistically. That is, the observed effects would be a remnant of learning how to read and write Arabic numbers and not a result of direct result of number word processing. Dotan (2023) provided evidence that language influences number processing very early during visual processing. When asked to name multi-digit numbers which were presented for 100 ms, Hebrew and Arabic speakers showed digit identification errors tied to their language’s number word order: Arabic speakers identified unit digits more accurately but decade digits less accurately than Hebrew speakers, reflecting visual scanning adapted to number word order. This might indicate that linguistic modulation of the UDCE is due to early visual analysis. On the contrary, Bahnmueller et al. (2019) argue that numbers are processed linguistically manipulating the UDCE through articulatory suppression (repeating “pataka”). By repeatedly saying “pataka”, linguistic processes remained occupied which eliminated the differences in the UDCE between German and English speakers. The authors argue that the linguistic modulation of the UDCE is therefore driven by the processing of number words, not merely the visual analysis of digits.

The French vigesimal number word system helps elucidate this, as each effect observed in multi-digit Arabic numeral processing may have a corresponding linguistic equivalent in the base-20 verbal structure. If the syntax of the French number word impacts the semantic processing of two-digit numbers, we hypothesize that there exists a base-20 equivalent of the UDCE in French participants for numbers above 60 where the base-20 system takes hold. For example, the number pair 74_86 is compatible in the regular base-10 system (74_86 because 7 < 8 and 4 < 6) but it is incompatible in the base-20 system (74_86 which reads as “60 14_80 6” and where 60 < 80 but 14 > 6). Henceforth, the proposed compatibility effect for numbers in the vigesimal system will be called the *unit-vigintade compatibility effect* (UVCE).

In addition to the UDCE, two-digit number comparisons also display a *within-decade effect*: The response to a number pair is faster when both numbers share the same decade (e.g., 35 vs. 37, decade: 3) compared to numbers with a decade distance of one (e.g., 35 vs. 47, decades: 3 vs. 4). Responses are faster as identical decade digits render the decade digits irrelevant and shift focus to the units, despite smaller overall distances in within-decade pairs (Huber et al., 2014). Similarly, in the French vigesimal system, numbers from 60–79 and 80–99 share the same *vigintade* (vigesimal equivalent of a decade), despite spanning over two decades. For instance, number pairs like 64 vs. 73 (pronounced “60–4” vs. “60–13”) share the same ‘decade word’, unlike 64 vs. 53 (pronounced “60–4” vs. “50–3”). Besides the within-decade effect, we hypothesized a *within-vigintade effect* where response times for between-decade pairs within the same vigintade are faster than for other between-decade pairs with equivalent decade distances. This would suggest that the decade word influences processing, creating a linguistic bias that prioritizes the verbal unit over numerical decade differences.

Both hypothesized linguistic effects, the UVCE and the within-vigintade effect, would allow us to examine the processing pathways that connect the visual input with the semantic representation. According to the most influential model of number processing, the Triple Code Model (Dehaene & Cohen, 1995, 1997), a given number’s semantic magnitude representation can be accessed via different pathways. Visual symbolic input such as Arabic digits can be transcoded directly via a pathway connecting the occipito-temporal visual number form area with the semantic number code in parietal cortex.

Alternatively, the parietal semantic code may be accessed through a *detour* via the verbal code that is processed in left-hemispheric, perisylvian language areas. In line with the latter, Dotan and Friedmann (2018) proposed a model for Arabic to verbal transcoding where numbers are first visually analyzed for identity, order and decimal structure to build a language-independent, visual number word frame, which is then passed to a second system which applies language-specific rules associated with their phonological and articulatory counterparts. In both cases, however, transcoding from visual to semantic magnitude code might take a detour via an intermediate linguistic step. Here, we take advantage of the dissociation between the visual code that adheres to the regular base-10 place-value system and the base-20 number word system in French. Finding these linguistic effects would imply that the semantic number code is accessed via the verbal number representation.

The current study aimed at investigating whether the semantic access to Arabic numerals involves linguistic modulation by using a classic two-digit number magnitude comparison task in French speakers. While Arabic numerals adhere to a culturally universal base-10 structure, French number words above 60 impose a base-20 logic - creating a natural dissociation between visual form and linguistic representation. If Arabic numbers are not processed linguistically, we should not find linguistic (i.e., vigesimal) counterparts to the UDCE and within-decade effect which are typically associated with the place-value structure of Arabic numerals. If we furthermore detect the proposed UVCE and within-vigintade effect, it will indicate that magnitudes are also activated via number word components. This would mean that verbal codes are spontaneously activated, producing a UVCE and a within-vigintade effect in addition to their decimal counterparts (i.e., the UDCE and the within-decade effect).

## METHODS

### Transparency and Openness

The study outline, data collection, and analysis plans were preregistered as part of a two-experiment study (https://aspredicted.org/qr6t-j3fr.pdf). For the sake of brevity, the results of the second experiment (bar task) will be reported elsewhere. We report how we determined our sample size, all data exclusions, manipulations, and measures in the study. All data, the analysis code, and research materials have been made publicly available on the Open Science Framework at https://osf.io/s5az3/?view_only=c3ac55083b5b41b5be69a0aaf96dea47. The study followed the latest version of the Code of Ethics of the World Medical Association (2013). All participants gave their informed consent before the study and received a compensation payment.

### Participants

Participants were recruited via prolific.com and pre-selected to be native French speakers who live and grew up in France—to make sure that participants were not, for instance, used to the Belgian or Swiss French number word syntax. Sixty-one participants (27 female) completed the study. One participant was excluded because fewer than 75% of their responses were correct. The remaining 60 participants were on average 28.67 years old (*Median* = 29, *SD* = 5.25). Even though the Prolific platform pre-selected participants to satisfy the aforementioned criteria, we asked a couple of control questions in a binary yes/no format (cf. Appendix A): 52 of 60 participants described themselves as right-handed, and none of the participants claimed not to count in French or to be bilingual. Participants had normal or corrected to normal vision and reported neither a previous history of neurological or psychiatric disorders nor difficulties in arithmetic.

### Design and Stimuli

Stimuli were created using the R programming language (R Core Team, 2021). To create a diverse set of stimuli with uncorrelated units and decades, we selected a set of tuples for units and for decades and then formed items from all possible combinations. For example, any decade tuple (3, 4) and a unit tuple (2, 7) would combine to form four different items: the base-10-compatible item 32_47, the incompatible item 37_42 as well as their reversed response sides 47_32 and 42_37. The decade tuples not only included the decade pairs of interest, i.e., numbers above 60 which are contained in the base-20 system but also pairs with a large decade distance (i.e., 30s vs. 80s and 40s vs. 90s). This way, we prevented the decades from being strongly correlated. If decade digits were too strongly correlated, participants might infer one number based on the other and not fully process each number which might discourage linguistic processing. The decade tuples were (3, 4), (3, 8), (4, 9), (5, 6), (5, 7), (6, 7), (6, 8), (6, 9), (7, 8), (7, 9), (8, 9). In the final stimulus set, decades were uncorrelated (|*r*| < .01) when within-decade items were not taken into account.

Unit tuples were chosen to keep a large unit-distance ensuring that we elicit a stronger UDCE (Macizo & Herrera, 2010). Additionally, we avoided numbers with the unit digit “1” because their French number words contain an additive conjunction (“…-et-un”) while the other numbers do not. The unit tuples were: (3, 4), (2, 7), (2, 8), (2, 9), (3, 9), (3, 8), (4, 7), (6, 7). Finally, we created items by combining unit and decade tuples and then excluding items in which a digit occurred more than once in an item. On top of these experimental items, 45.25% of number pairs were within-decade items (both decades were equal) to ensure that the unit-decade compatibility effect was elicited (Macizo & Herrera, 2011). To achieve this ratio of within-decade items (around 40%–50%), unit tuples were combined with decades ranging between 2 and 9. The unit tuples for within-decade trials were (3, 4), (2, 7), (2, 8), (2, 9), (3, 6), (3, 8), (3, 9), (4, 9), (5, 8), (6, 7). Also here, within-decade items were excluded when one of the units was equal to the decades. In total, this resulted in 358 stimuli which are further described in Appendix B.

### Procedure

Participants were directed to the consent form by Prolific and asked again to only participate if French (as spoken in France) was their mother tongue. If they consented to participate, they completed a short form with control questions about language and math usage (Appendix A).

After that, they were introduced to the number comparison task. Each trial started with a fixation cross of 500 ms. Then two two-digit numbers appeared side-by-side with the height of 6 cm and the width of 3 cm in “Courier New” font. The gap between the two numbers on the center of the screen was 3 cm. The participant had a maximum of 3,000 ms to indicate which of the two numbers was greater in magnitude by pressing either one of two buttons. Pressing the button “S” indicated that the left number was greater, and pressing “K” indicated that the right number was greater. Once the participant had released the “S” or “K” key, they moved on to the next trial irrespective of the 3,000 ms being over or not.

Participants completed a practice loop of six practice trials which let them continue as long as fewer than three of the six responses were incorrect. After each trial in the practice loop, short feedback appeared for 1,000 ms if their response was correct, incorrect, or too slow (slower than 3,000 ms). If they continued to make mistakes, a clarification of the instructions appeared. Once participants completed the practice loop, they continued to complete the number comparison task. The task itself lasted roughly 13 minutes when excluding breaks which were prompted every 60 trials. The experiment was programmed in HTML, CSS and JavaScript.

### Analyses

The analyses were conducted using the R Statistical Software (R Core Team, 2021). For analyses of response times, we excluded responses faster than 200 ms, slower than three standard deviations above the participant’s mean, or incorrect responses. Overall, we excluded 6.34% of all responses. To test the proposed UVCE as well as the within-vigintade effect, we ran linear mixed models using the “lme4” package in R (Bates et al., 2015). In addition, we compared the within-vigintade items to within-decade items as well as other between-decade items with the same decade distance by applying paired *t*-tests to participant-wise averages.

We substantiated null effects by applying Bayesian *t*-tests with R’s “BayesFactor” package (Morey et al., 2024). The Bayesian *t*-tests assumed a Cauchy prior distribution with a scale parameter of $\frac{\sqrt{2}}{2}$ which is the package’s default scale parameter for the prior distribution. Bayes factors were interpreted as recommended by Jeffreys (1998).

## RESULTS

Descriptive statistics of task performance (RT) are provided in Appendix B. Our first analysis aimed at investigating the UDCE and UVCE. To this end, we considered all trials that qualify to be compatible or incompatible in a base-10 or base-20 manner and excluded within-decade and within-vigintade items (60s vs. 70s, 80s vs. 90s) from the analysis. We included as predictors unit-decade incompatibility (yes: 1, no: 0; unit-decade incompatibility) and a predictor for both numbers being larger than 60 (yes: 1, no: 0) because that is when the base-20 system begins in two-digit numbers. This factor is important for two reasons: First, this factor captures potential problem size effects (slower responses the larger the numbers involved). Second, we do not want effects to be explained by the fact that numbers within the base-20 system are generally processed differently. Furthermore, we included the UVCE: Numbers above 60 can be either unit-vigintade compatible (dummy-coded as 0) or unit-vigintade incompatible (dummy-coded as 1). To also account for potential ceiling effects, we included an interaction term between unit-decade and unit-vigintade incompatibility (UDCE, UVCE). If an item is unit-decade as well as unit-vigintade incompatible, maybe the effects are not additive but there might be a ceiling at which unit-decade or unit-vigintade incompatibility no longer affects response times. Table 1 provides the model’s estimates, confidence intervals, and *p*-values for all number comparisons.

**Table 1.** Linear mixed model results for all numbers.

**Variable**	**Estimate (*β*)**	**CI**_**95%**_	***p***
*SD*_*participant*_	107.26	[87.94, 126.58]	---
*SD*_*residual*_	151.37	[149.15, 153.59]	---
intercept	699.71	[672.29, 727.13]	<.001
UD-incompatible	55.74	[47.64, 63.83]	<.001
numbers > 60	−26.08	[−35.52, −16.63]	<.001
numbers > 60: UV-incompatible	45.77	[28.04, 63.50]	<.001
numbers > 60: UD-incompatible	−7.24	[−24.00, 9.52]	.397
numbers > 60: UV-incompatible × UV-incompatible	−9.92	[−33.33, 13.49]	.406

*Note*. All items were included except for within-decade and within-vigintade items; UD: unit-decade (base-10), UV: unit-vigintade (base-20); *p*-values were estimated with the R package “lmerTest” (Kuznetsova et al., 2017).

Unit-decade incompatible trials slowed responses down by roughly 56 ms. This effect was not modulated when numbers were in the base-20 system (i.e., greater than 60). Also, responses were faster when both numbers were larger than 60 (i.e., entering the base-20 system). However, when numbers were larger than 60, a UVCE existed: base-20-incompatible items elicited slower responses than base-20-compatible items (*β* = 45.77 ms). In sum, the model indicates not only a UDCE for numbers larger and smaller than 60 but also a UVCE for numbers larger than 60.

**Figure 1.**
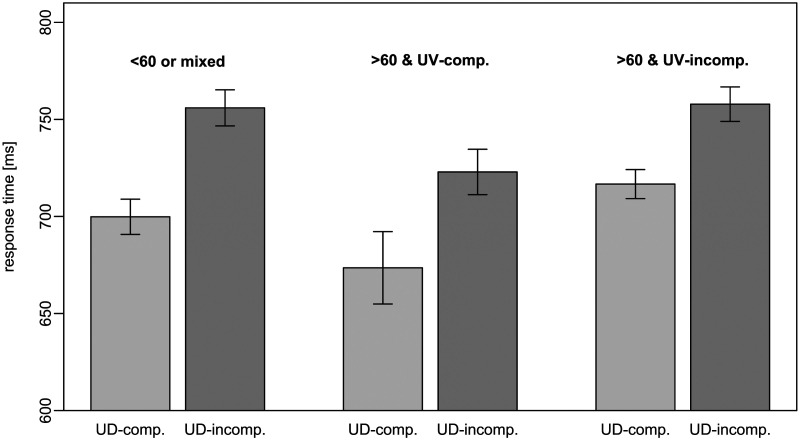
Average Response times in unit-decade compatibility vs. unit-vigintade compatibility for numbers larger than 60. *Note*. We observed both a UDCE as well as a UVCE compatibility effect but no interaction; UD: unit-decade, UV: unit-vigintade; error bars depict confidence intervals (95%) that have been corrected for between-subject variance according to Morey (2008) with the “papaja” package (Aust & Barth, 2023) to allow for within-subject comparisons of the confidence intervals; data from number pairs with both numbers larger than 60 or any other number pairs are shown; within-vigintade items (60s vs. 70s and 80s vs. 90s) were excluded for they are neither unit-vigintade-compatible nor unit-vigintade-incompatible.

To check the robustness of the results, we ran a second model only consisting of items where both numbers were larger than 60. This way, the potential influence of problem size was removed, and all numbers were expressed in a base-20 manner. The variables were identical to the model displayed in Table 1 except that the variable indicating if both numbers were larger than 60 was now redundant. Table 2 shows that both UDCE and UVCE again significantly predicted RT, indicating a better task performance in unit-decade compatible (*β* = 33.01, *p* < .001) and unit-vigintade compatible number pairs (*β* = 28.72, *p* < .001) than in incompatible ones. The average response times for trials with both numbers being larger than 60 are presented in Figure 1.

**Table 2.** Linear Mixed model results for numbers larger than 60.

**Variable**	**Estimate (*β*)**	**CI**_**95%**_	***p***
*SD*_*participant*_	109.18	[89.38, 128.98]	---
*SD*_*residual*_	150.83	[148.07, 153.58]	---
intercept	690.82	[662.9, 718.74]	<.001
UD-incompatible	33.01	[22.27, 43.76]	<.001
UV-incompatible	28.72	[11.76, 45.67]	<.001
UD-incompatible × UV-incompatible	19.37	[−1.13, 39.87]	.064

*Note*. UD: unit-decade (base-10), UV: unit-vigintade (base-20); *p*-values were estimated with the R package “lmerTest” (Kuznetsova et al., 2017).

Yet, the UVCE suffers from language-inherent limitations. The base-20 system only occurs in the French numbers larger than 60. This means that only four decades can be exploited to measure this effect—and only two base-20 decades (“vigintades”). While the UDCE can be balanced in terms of unit and decade distance by switching the units of a number pair, the UVCE cannot be manipulated like that. Table 3 presents two constraints for studying the UVCE: First, some conditions of the UVCE are only true for one pair of decades; for example, only the comparisons of 70s vs. 80s qualify to be unit-decade compatible and unit-vigintade incompatible. Secondly, these four conditions also differ in their average decade distance—these effects are naturally confounded with the UVCE. Therefore, the UVCE alone does not suffice to show that language influences the processing of Arabic numbers.

**Table 3.** Possible decade pairs for detecting the UVCE in the French language.

	**Unit-vigintade compatible**	**Unit-vigintade incompatible**
unit-decade compatible	60s–80s: 2	70s–80s: 1
	70s–90s: 2	
	60s–90s: 3	
unit-decade incompatible	60s–90s: 3	70s–80s: 1
		60s–80s: 2
		70s–90s: 2

*Note*. Number pairs are represented by the smaller and larger decade and the distance between the decade digits; notice that all possible decade pairs are included except for the 60s–70s and 80s–90s because they are within-vigintade items (the base-20 equivalent of within-decade items).

This is why we also investigated other aspects of the base-20 system by focusing on the base-20 equivalent of within decade items, i.e., within-vigintade items. For the analysis of within-vigintade trials, we excluded all trials with a decade distance larger than one. We distinguished three groups of stimuli: within-decade items (e.g., 61 vs. 68), within-vigintade items (e.g., 61 vs. 78) which share the same vigintade word, and other items with a decade distance of one (e.g., 51 vs. 68) which do not share the same decade word. Linear mixed models revealed a significant within-decade effect and a significant within-vigintade effect. These results are presented in Table 4.

**Table 4.** Linear mixed model for numbers with a decade distance of 0 or 1 testing the within-vigintade and within-decade effects.

**Variable**	**Estimate**	**CI**_**95%**_	***p***
*SD*_*participant*_	113.83	[93.45, 134.22]	---
*SD*_*residual*_	154.36	[152.56, 156.16]	---
intercept	765.85	[736.84, 794.87]	<.001
within decade	−21.45	[−27.80, −15.10]	<.001
within vigintade	−19.45	[−28.21, −10.70]	<.001

*Notes. p*-values were estimated with the R package “lmerTest” (Kuznetsova et al., 2017).

**Figure 2.**
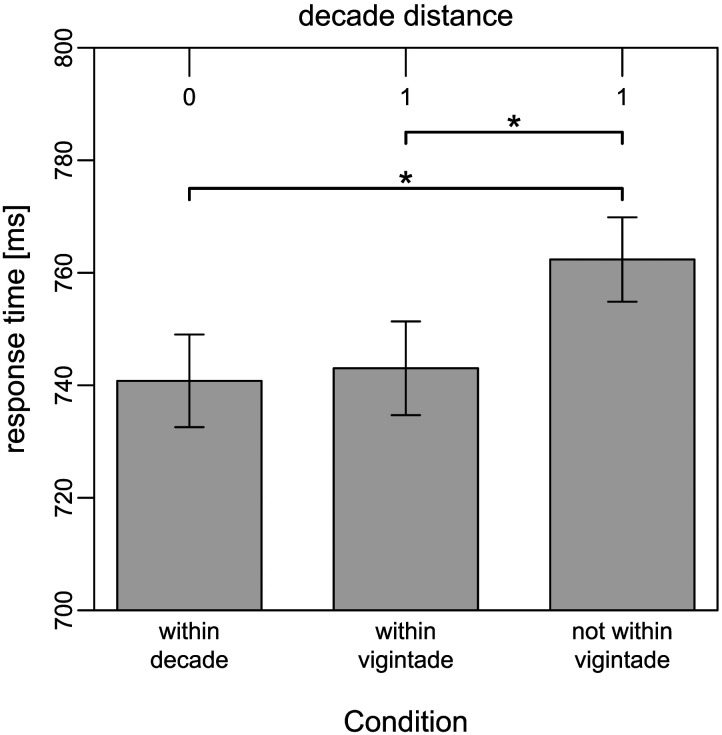
Response times of within-decade items, within-vigintade (between-decade) items, and between-decade (and between-vigintade) items. *Note*. Even though decade distance (top axis) is 1, response times to between-decade items from the same vigintade (“within-vigintade items”; e.g., 62 vs. 73) do not significantly differ from within-decade items (e.g., 62 vs. 63); in contrast, between-decade items from the same vigintade (e.g., 62 vs. 73) are responded to significantly faster than between-decade items from a different vigintade (e.g., 72 vs. 83) although decade distance is always 1; error bars depict confidence intervals (95%); to adequately interpret confidence intervals, they were corrected for between-subject variance according to Morey (2008) with the “papaja” package (Aust & Barth, 2023); * : *p* < .001.

Not only were within-decade items responded to faster than between-decade items with a decade distance of one (*β* = −21.45, *p* < .001) but so were within-vigintade items (*β* = −19.45, *p* < .001) which share the same vigintade word but different decade digits. Average response times are presented in Figure 2. The estimates for within-decade and within-vigintade items indicate that they might be responded to equally fast. To test these differences, we ran additional frequentist and Bayesian *t*-tests. Within-decade trials had faster responses than trials with a decade distance of one, *t*(59) = 3.87, *p* < .001, Δ = 21.49 ms, BF_10_ = 87.10 ± 0%. The Bayes factor indicates very strong evidence for the within-decade effect. Responses to within-vigintade trials (60s vs. 70s, 80s vs. 90s) were also faster than other trials with a decade distance of one, *t*(59) = 3.36, *p* = .001, Δ = 18.84 ms, BF_10_ = 20.33 ± 0%. Here the Bayes factor indicates strong evidence for the within-vigintade effect. Within-vigintade items and within-decade items did not differ significantly in their response times, *t*(59) = −0.43, *p* = .666, Δ = 2.65 ms, BF_10_ = 0.15 ± 0.08%. Because the Bayes factor is between 0.33 and 0.10, it indicates moderate evidence in favor of the null hypothesis, supporting the claim that within-vigintade and within-decade items were processed at the same speed.

However, since numbers below 60 were among the control items (in within-decade as well as non-within-vigintade items), the within-vigintade effect could also be explained by problem size or an overall difference between the base-10 and base-20 number word systems. To address the general influence of the verbal number systems above and below 60, we fit another mixed model identical to the one presented in Table 4 except for adding a dummy variable indicating whether numbers were larger than 60, i.e., whether numbers were in the range of the base-20 number word system (yes: 1, no: 0). Any potentially significant within-vigintade cannot be explained by problem size or an overall difference between the number word systems above or below 60. The results of this linear mixed model are presented in Table 5. Although number pairs above 60 were responded to faster than other number pairs (*β* = −6.84, *p* = .019) the within-vigintade effect remained significant (*β* = −14.07, *p* = .005).

**Table 5.** Within-vigintade and within-decade effect accounting for numbers larger than 60.

**Variable**	**Estimate**	**CI**_**95%**_	***p***
*SD*_*participant*_	113.82	[93.43, 134.20]	---
*SD*_*residual*_	154.33	[152.53, 156.13]	---
intercept	767.31	[738.27, 796.34]	<.001
within decade	−19.22	[−25.83, −12.60]	<.001
within vigintade	−14.07	[−23.92, −4.22]	.005
numbers > 60	−6.84	[−12.57, −1.11]	.019

*Notes. p*-values were estimated with the R package “lmerTest” (Kuznetsova et al., 2017).

Finally, if within-vigintade trials are processed similarly to within-decade trials, we would expect a smaller UDCE in within-vigintade trials compared to other trials with a decade distance of one. Within-decade items have a processing advantage because recognizing that decade digits are identical (e.g., via visual matching) frees up processing resources that can be attributed to the semantic elaboration of the unit digits (Huber et al., 2014). Similarly, within-vigintade items may shift the focus towards the verbal unit (ranging 1–19) since the vigintade word is identical in these items. Indeed, within-vigintade items displayed a smaller UDCE (*M* = 52 ms) compared to other trials with a decade distance of one (*M* = 67 ms), a one-tailed *t-*test revealed, *t*(60) = −1.79, *p* = .039, Δ = 15 ms.

## DISCUSSION

The current study aimed to test the linguistic relativity hypothesis by exploiting the discrepancies between the Arabic number notation and the French number word syntax. The way an Arabic numeral is understood might not only involve recognizing its visual form but also involve processing it through its verbal representation. If Arabic numbers are processed linguistically to some extent, we might be able to detect signs of specific syntactical rules that govern how number words are constructed. We therefore not only expected the symbolic UDCE and within-decade effect but also expected their linguistic equivalents: a UVCE and within-vigintade effect.

First, the UDCE for numbers existed not only for numbers smaller than 60 but also larger than 60. This means that the Arabic digits within the place-value structure activated magnitude representations irrespective of how the number word is constructed. These effects suggest that the digits are processed within the place-value structure in which Arabic numbers are written. But our data also show that number words were accessed even though numbers were presented as Arabic numerals. The models revealed a UVCE for numbers larger than sixty which was of a roughly equivalent size of the UDCE. This indicates that the number word was processed, and the number’s constituting unit and decade words were compared independently from the digits’ magnitudes of the number.

One may argue that the UVCE might be due to its natural confound with decade distance. However, our analyses of the within-vigintade effect provided further evidence for base-20 influences in two-digit magnitude comparison. Number pairs sharing the same vigintade word in the French vigesimal system (i.e., 60s vs. 70s or 80s vs. 90s, within-vigintade pairs) were processed more rapidly than other between-decade pairs with equivalent decade distances (e.g., 50s vs. 60s). Problem size or the overall influence of the base-20 system for numbers above 60 did not account for this within-vigintade effect. This strengthens the claim that two-digit numbers are at least by some degree compared based on their verbal number word representations.

The vigintade words in the French vigesimal system (e.g., “soixante” for 60s and 70s, “quatre-vingt” for 80s and 90s) likely elicit processing patterns analogous to those observed in within-decade pairs (e.g., 50s vs. 50s). Akin to how the focus shifts to the unit digits in within-decade comparisons (Huber et al., 2014), participants might also prioritize the verbal unit in within-vigintade items. We consequently hypothesized that the unit-decade compatibility effect (UDCE) would be attenuated in within-vigintade pairs, as participants may prioritize the verbal unit. Consistent with this hypothesis, the UDCE was significantly reduced in within-vigintade pairs compared to other between-decade pairs with the same decade distance. Such reduction in the UDCE lends support to the idea that numbers were compared by their number word components (decade and unit word) and not merely by their Arabic digits. These findings cannot be explained by decade distance and corroborate the hypothesis that the vigesimal structure of the French number words impacts the magnitude comparison of Arabic numerals.

Taken together, our results indicate that numbers are not only processed within their place-value structure of their constituting digits, but also by their number word components. While the UVCE comes with limitations, these limiting factors do not extend to the within-vigintade effect. Because the number’s vigintade word is the same in both numbers of a within-vigintade item, they might be processed like within-decade items where the numbers’ decade words as well as the decade digits are identical.

Although the magnitude comparison task’s stimuli were Arabic digits and therefore only required symbolic digit processing, the emergence of vigesimal effects suggests that verbal codes are activated automatically, conceivably introducing interference from conflicting verbal magnitude cues (e.g., “60–12” vs. 72). This aligns with unintentional numerical extraction in Stroop-like paradigms (Huang et al., 2021) and might extend to verbal transcoding pathways. Supporting evidence for spontaneous verbal activation comes from studies on bilinguals. Salillas and Carreiras (2014) found that Spanish-Basque bilinguals who learned math in Basque, a language that consistently displays vigesimal number words, showed distinct EEG patterns during two-digit number magnitude comparisons. Such EEG patterns indicate distinct spontaneous recruitment of linguistic processing. The co-occurrence of decimal effects (UDCE, within-decade effect) and vigesimal effects (UVCE, within-vigintade effect) in our results further supports that verbal activation at least operates in parallel with direct symbolic-to-magnitude transcoding (Bahnmueller et al., 2019). While the extent of automaticity remains to be quantified, e.g., by applying imaging techniques, our findings confirm that Arabic numerals are at least partially and spontaneously processed via language-specific verbal pathways.

### Multiple Pathways to Number Magnitude

So far, it has been assumed that numbers are primarily processed following their Arabic digit notation by combining the digits’ magnitudes with their place-value information (e.g., Nuerk et al., 2011). However, having revealed traces of the vigesimal system, our data suggest that numbers might also be processed linguistically. In line with the Triple Code Model (Dehaene & Cohen, 1995, 1997), multi-digit numbers might not only be directly transcoded from their visual code into the magnitude code but also indirectly via a *verbal detour*: The traces of the vigesimal system suggest that numbers might also be transcoded from their visual code into their verbal code and then into their magnitude code. This means that an Arabic numeral (such as 72) activates the number word (pronounced “60–12”) which, then again, activates the magnitude code (“60” and “12”). Crucially, this activation of the number word is not part of the two-digit number’s Arabic code (“70” and “2”) and independent from the overall magnitude of the number (72).

A *verbal detour* explanation not only accords with the Triple Code Model’s assumptions of independent number codes, but it also aligns with a growing body of evidence for multiple transcoding pathways from visual codes to magnitude codes. Santens et al. (2010) provide neural support for such pathway diversity. They showed that non-symbolic quantities engage an intermediate number-sensitive stage in the posterior superior parietal cortex towards the intraparietal sulcus, whereas symbolic digits bypass this stage via a direct pathway. Extending this framework, our data reveal an additional verbal mediation route for Arabic numerals suggesting that language-specific structure can recruit verbal codes even when task demands favor magnitude-based comparison. Thus, visual-to-magnitude transcoding may proceed via at least three pathways: a direct symbolic route, a number-sensitive summation route for non-symbolic input, and a language-dependent verbal route for symbolic numerals. While Santens et al. (2010) examined symbolic single-digit numbers, the current study extends the approach to multi-digit numbers which require additional place-value processing. The activation and processing of place-value information might require additional processing steps which could be facilitated by linguistic processes. Particularly when multi-digit symbolic numbers need to be identified and activated, a detour via the verbal code could be beneficial since processing through the verbal code could be particularly beneficial in various everyday tasks (e.g., in arithmetic fact retrieval or in parity judgment; Bahnmueller et al., 2018). However, since not only the symbolic UDCE but also the linguistic UVCE seem to co-exist for numbers above 60 in French speakers, we suggest that both the direct route as well as the indirect asemantic verbal route might be used in parallel for two-digit number comparisons.

Dotan (2023) proposes an alternative explanation to the verbal detour, namely that verbal processes exert top-down modulation on early visual digit analysis. In a brief-exposure (100 ms) number naming task, Arabic speakers (inverted word order) identified the unit digit slightly earlier and the decade digit slightly later than Hebrew speakers, showing that spoken-word order can reshape the visual analyzer’s serial scanning at a pre-semantic stage. However, these indications of early visual influences on digit scanning are based on the incongruence between digit order and spoken-word order in Hebrew, which does not exist in French. Consequently, early top-down visual effects cannot account for the vigesimal patterns observed here. These effects are most parsimoniously explained by spontaneous activation of the verbal code and parallel comparison of its components. Given the current findings, it is likely that both early top-down influences from the visual analyzer and later verbal mediation occur in the processing of Arabic numerals.

Another alternative to the verbal-detour account might be that number words become incorporated into the core symbolic processing system itself over the course of development, gradually blurring the distinction between an independent visual code and a verbal code. Through repeated co-activation of verbal and symbolic representations, language-specific syntax (e.g., the French base-20 system) might be gradually embedded into the symbolic processing system itself (Skagenholt et al., 2021). Supporting evidence includes the age-related increase of the decimal UDCE (Nuerk et al., 2004) and its cross-linguistic modulation (Moeller, Shaki, et al., 2015; Pixner, Zuber, et al., 2011). Although the verbal-symbolic integration account is consistent with developmental and cross-linguistic evidence, it posits a deeper blending of visual and verbal number codes across development than is assumed by the Triple Code Model (Dehaene & Cohen, 1995, 1997). According to that view, Arabic number processing would involve broader asemantic visuo-verbal representations instead of a verbal detour. Future neuroimaging research should aim at clarifying how language is involved with number processing.

### Linguistic Relativity vs. Universality

The Language of Thought hypothesis (Fodor, 1975), a sub-theory of the cultural universality perspective, argues that core cognitive concepts like numerosity are innate and transcend linguistic boundaries, while language merely serves as a communicator of these language-independent ideas. This hypothesis proposes an internal, language-independent mental code where complex representations arise from the recursive composition of discrete symbols into programs. Dehaene et al. (2022) extend this to a neuroscientific context, suggesting that humans possess multiple domain-specific languages of thought (e.g., for shapes, sequences, or mathematics) distinct from natural language processing. Applied to numbers, Pajot et al. (2025) use frequencies of number words across languages in large text corpora to argue that exact number concepts emerge universally from compositional algebraic operations (addition and multiplication) on primitive symbols. This number frequency framework predicts that number processing should remain unmodulated by language-specific syntax, attributing frequency patterns to psychological simplicity.

Our results found not only the symbolic UDCE and within-decade effect but also equivalent effects originating in the number word syntax. These linguistic influences contradict the Language of Thought approach brought forward by Dehaene et al. (2022). Our results instead favor the Linguistic Relativity Hypothesis (Hoijer, 1954). Linguistic relativity posits that the syntactical and lexical properties of a language actively shape cognitive concepts, including those related to numbers. According to linguistic relativity, language does not merely label language-independent ideas but influences how speakers perceive and process reality. Unlike Language of Thought approaches, linguistic relativity predicts that the vigesimal structure in French for numbers above 60 should modulate numerical cognition, leading to language-specific effects in tasks like number magnitude comparison. Despite Arabic numerals being identical across cultures and thus language-neutral, and despite number words being task-irrelevant, their traces reveal linguistic detours rather than direct symbol-to-magnitude mapping.

This aligns with experimental evidence of linguistic influences (e.g., Bahnmueller et al., 2019; Dotan, 2023) and challenges Pajot et al.'s (2025) Language of Thought model, as their frequency analyses do not account for syntax-driven differences of number words. This begs the question of why vigesimal structures would affect behavioral outcomes if number concepts were independent of language. Our findings indicate partial linguistic processing of Arabic numerals via a verbal detour, evidenced by the persistence of a decimal UDCE and within-decade effect alongside their vigesimal counterparts. In this sense, language plays a central role in how numbers, and potentially other concepts, are conceived.

### Conclusion

In the current study, we not only found a UDCE in French numbers below and above 60, but also verbal equivalents in numbers above 60. The UVCE indicates that the semantic code of Arabic numbers was accessed via a verbal route. While there are some limitations to the interpretation of the UVCE, further concordant evidence came from within-vigintade items. Whenever between-decade items with a decade distance of one were within-vigintade items, they were processed as quickly as within-decade items. Meanwhile, responses to other between-decade items with a decade distance of one were significantly slower. These findings cannot be explained by processing via symbolic-to-magnitude pathways alone. We propose that there exists also a language-dependent verbal detour that recruits number word syntax even in silent magnitude comparison. The coexistence of decimal and vigesimal effects suggests these routes operate in parallel, with verbal mediation neither obligatory nor dominant but culturally shaped and developmentally integrated (Nuerk et al., 2004; Skagenholt et al., 2021).

The engagement of verbal number codes challenges Language-of-Thought accounts positing a purely mathematical compositionality of number concepts (Dehaene et al., 2022; Pajot et al., 2025). The linguistic traces in number processing support linguistic relativity (Hoijer, 1954): French speakers’ processing of Arabic numerals bears the imprint of their verbal base-20 system, demonstrating that language does not only label, but actively scaffold numerical cognition. Far from being language-neutral glyphs, Arabic numerals are processed through a hybrid symbolic-linguistic lens.

## ACKNOWLEDGMENTS

We would like to thank Natalia Hacquart and Claire Pruvot for their invaluable administrative support.

## FUNDING INFORMATION

This work was supported by the Agence Nationale de la Recherche (ANR-22-CE28-0020 and ANR-23-FRAL-0008).

## AUTHOR CONTRIBUTIONS

R.J.: Conceptualization; Data curation; Formal analysis; Investigation; Methodology; Visualization; Writing – original draft. A.K.: Conceptualization; Supervision; Writing – original draft; Writing – review & editing. A.V.: Conceptualization; Writing – review & editing. E.K.: Conceptualization; Funding acquisition; Methodology; Resources; Supervision; Writing – original draft; Writing – review & editing.

## OPEN DATA

Data and analysis scripts have been stored in an OSF repository at https://osf.io/s5az3/?view_only=bfbaa51f5cf045938b622a0c50be1967 (non-blinded access).

## APPENDIX A

**Table A1.** Descriptive statistics of the pre-study questionnaire.

Question in French	Translation to English	Response type	Response (*N* = 60)
Êtes-vous gaucher ou droitier ?	Are you left-handed or right-handed?	radio	right: 52; left: 5; both: 3
		button	
Avec quelle main écrivez-vous ?	Which hand do you write with?	radio button	right: 53; left: 7; both: 0
Ma vue est claire ou je porte des lunettes pour que la vue soit corrigée.	My vision is clear, or I wear eyeglasses to correct my vision.	checkbox	agreed: 60
Je souffre d’un trouble neurologique ou psychiatrique qui a été confirmé par un médecin.	I have a neurological or psychiatric disorder that has been confirmed by a physician.	checkbox	agreed: 0
Je suis dyslexique.	I am dyslexic.	checkbox	agreed: 0
Je suis dyscalculique.	I am dyscalculic.	checkbox	agreed: 0
Le français est ma langue maternelle.	French is my mother tongue.	checkbox	agreed: 60
J'ai grandi dans un foyer bilingue.	I grew up in a bilingual household.	checkbox	agreed: 7
Je parle couramment une autre langue.	I fluently speak another language.	checkbox	agreed: 36
J'ai grandi en France pendant la majeure partie de mon enfance.	I grew up in France during most of my childhood.	checkbox	agreed: 55
Je compte les objets et fais des calculs dans ma tête en français.	I count objects and do mental arithmetic in French.	checkbox	agreed: 54
J'ai des difficultés avec le calcul mental.	I have problems with mental arithmetic.	checkbox	agreed: 8
J'ai des problèmes avec la grammaire française.	I have problems with French grammar.	checkbox	agreed: 2
J'ai des problèmes d'orthographe.	I have problems with spelling.	checkbox	agreed: 1
J'ai lu un livre du début à la fin au cours des deux dernières années.	I read a book from cover to cover in the last two years.	checkbox	agreed: 51

*Note*. Except for the radio button questions, questions were asked on a voluntary basis which might explain why some participants did not agree on some items.

## APPENDIX B

**Table B1.** Summary statistics for stimulus matching and response times.

		Descriptive statistics			responses
		*M* (*M*_*log*_)			*M* (*SD*)
	Item group	unit distance	decade distance	total distance	RT
both numbers > 60	b10 comp. & b20 comp.	4.15	2.38	28.00	674
		(1.23)	(0.85)	(3.32)	(102)
	b10 comp. & b20 incomp.	4.67	1.00	14.67	717
		(1.25)	(0.00)	(2.67)	(130)
	b10 incomp. & b20 comp.	4.00	3.00	26.00	723
		(1.22)	(1.10)	(3.26)	(121)
	b10 incomp. & b20 incomp.	4.36	1.73	12.91	758
		(1.23)	(0.50)	(2.42)	(116)
	b10 comp. & within b20	3.89	1.00	13.89	721
		(1.09)	(0.00)	(2.62)	(114)
	b10 incomp. & within b20	3.89	1.00	6.11	775
		(1.09)	(0.00)	(1.74)	(107)
	within b10	4.05	0.00	4.05	742
		(1.20)	(undefined)	(1.20)	(116)
rest	b10 comp.	4.67	2.54	20.83	700
		(1.35)	(0.68)	(2.56)	(104)
	b10 incomp.	4.53	2.54	30.00	756
		(1.32)	(0.68)	(3.23)	(114)
	within b10	4.05	0.00	4.05	747
		(1.21)	(undefined)	(1.21)	(115)

*Note*. Cells contain the mean distance and the mean of log-distance in parentheses;

b10: base-10, b20: base-20; “within b20” items are within-vigintade items; “within b10” items are within-decade items;

mean unit distance ranged between 3.89 and 4.67;

RT: response times and their *SD* refers to between-subject variance.

## APPENDIX C

Here, we intend to check the robustness of the within-vigintade effect. We not only ran different linear mixed models but also *t*-tests. Again, we distinguished three groups of stimuli: within-decade items (e.g., 61 vs. 68), within-vigintade items (e.g., 61 vs. 78) which share the same vigintade word, and other items with a decade distance of one (e.g., 51 vs. 68) where both numbers do not have the same decade word. Linear mixed models revealed a significant within-decade effect and a significant within-vigintade effect. These results are presented on the left side of Table C1.

However, when we isolate numbers above 60 and run the same tests, none of these differences remained significant (right side in Table 4): Neither within-vigintade trials nor within-decade trials were significantly faster than trials with a decade distance of one. Because we suspected these differences to be due to a lack of statistical power, we ran additional Bayesian *t*-tests to test the strength of the null hypothesis: there was moderate evidence against the within-decade effect, *t*(59) = 0.98, *p* = .330, Δ = 8.66 ms, BF_10_ = 0.22 ± 0.06%, and against the within-vigintade effect, *t*(59) = 0.45, *p* = .653, Δ = 3.78 ms, BF_10_ = 0.16 ± 0.08%. The removal of numbers below 60 likely underpowered this sensitivity check for two reasons: First, the within-decade effect, typically significant in two-digit number comparisons (e.g., Huber et al., 2014), was not significant here. Second, restricting comparisons to numbers above 60 limits non-within-vigintade items to the 70s-80s number pairs, drastically reducing the item pool. With small response time differences, the null model fit better in the Bayesian *t*-tests, leading to moderate evidence for the null hypotheses. The mixed model presented in Table 5 addresses these issues by including “numbers > 60” in the model. We could therefore control for the number word system (base-10 vs. base-20 verbal number system). The within-vigintade effect remained significant.

**Table C1.** Two linear mixed models investigating the within-vigintade effect considering either all numbers or items in which both numbers were above 60.

	**All numbers**			**Only numbers above 60**		
**Variable**	**Estimate**	**CI**_**95%**_	***p***	**Estimate**	**CI**_**95%**_	***p***
*SD*_*participant*_	113.833	[93.45, 134.22]	---	112.01	[91.79, 132.22]	---
*SD*_*residual*_	154.360	[152.56, 156.16]	---	155.38	[152.89, 157.86]	---
intercept	765.852	[736.84, 794.87]	<.001	152.31	[721.66, 782.76]	<.001
within decade	−21.451	[−27.80, −15.10]	<.001	−10.19	[−22.96, 2.58]	.118
within vigintade	−19.453	[−28.21, −10.70]	<.001	−6.05	[−19.88, 7.79]	.392

*Note*. *p*-values were estimated with the R package “lmerTest” (Kuznetsova et al., 2017).
